# Research progress of stem cells in the treatment of atherosclerosis

**DOI:** 10.3389/fcell.2025.1722416

**Published:** 2026-01-02

**Authors:** Peifei Shi, Chao Ren, Hongjie Tong

**Affiliations:** 1 Department of Intensive Care Unit, Affiliated Jinhua Hospital, Zhejiang University School of Medicine, Jinhua, China; 2 Department of Hepatobiliary and Pancreatic Surgery, Affiliated Jinhua Hospital, Zhejiang University School of Medicine, Jinhua, China

**Keywords:** atherosclerosis, stem cell therapy, induced mesenchymal stem cells, pluripotent stem cells, vascular repair, plaque stabilization

## Abstract

Atherosclerosis (AS) is the primary pathological basis for the disability and mortality rates of global cardiovascular diseases. Its core characteristics are abnormal deposition of blood vessel wall lipids, chronic inflammatory activation, and vascular structural remodeling, which ultimately lead to acute cardiovascular and cerebral vascular events such as coronary heart disease and cerebral infarction. Existing treatment methods, such as statins and interventional interventions, can only delay disease progression and cannot reverse the pathological damage to blood vessels that has already occurred. Stem cells provide a novel strategy for the targeted therapy of AS due to their multi-directional differentiation potential, immune regulatory ability, and tissue repair properties. This review systematically reviews the research progress of stem cells in the treatment of AS in recent years, focusing on the mechanism of the main cell types such as mesenchymal stem cells (MSCs), induced pluripotent stem cells (iPSCs), and endothelial progenitor cells (EPCs), including regulating lipid metabolism, inhibiting inflammatory reaction, repairing vascular endothelium, and stabilizing atherosclerotic plaque. This study summarizes the key evidence from animal experiments and clinical trials in 2023–2025; analyzes core challenges such as low homing efficiency, short survival time, and the risk of immune rejection of stem cells; and proposes optimization strategies such as gene modification, biomaterial carriers, and combination therapy. Finally, the application prospects of single-cell sequencing, organoid models, and precision delivery systems in promoting the clinical translation of stem cells are discussed, with specific implementation paths being supplemented: single-cell sequencing can analyze the heterogeneity of stem cells in the AS lesion microenvironment (e.g., subtype differentiation differences of MSCs under hypoxic conditions) to screen high-activity stem cell subpopulations; vascular organoids constructed from patient-derived iPSCs can simulate the *in vivo* lipid deposition-inflammatory microenvironment to evaluate stem cell therapeutic effects; and precision delivery systems can enhance lesion targeting via ligand modification (e.g., anti-VCAM-1 antibody-modified PLGA carriers), thus providing theoretical basis and research directions for the disease modification therapy of AS.

## Introduction

Atherosclerosis (AS), a degenerative disease with chronic inflammation of the blood vessel wall and lipid metabolism disorder as its core, has become the primary cause of ischemic heart disease (IHD) and ischemic stroke worldwide (Jee Yeon et al., 2023). According to the latest research, the number of deaths caused by AS-related cardiovascular events worldwide exceeds 17.9 million annually, accounting for 19.2% of the total deaths (Yuta et al., 2022). The pathological process of AS develops gradually, from the early formation of lipid streaks (deposition of low-density lipoprotein cholesterol under the endothelium) to the medium-term aggregation of foam cells (formed after phagocytosis and oxidation of low-density lipoprotein by macrophages) and the release of inflammatory factors, and it finally develops into vulnerable plaques with thinner fibrous caps and enlarged lipid cores (Luca et al., 2022; Federica et al., 2023). Thrombosis caused by plaque rupture is the direct cause of acute myocardial infarction and cerebral infarction (Xiang-Rui et al., 2023).

The existing clinical treatment plans focus on “controlling risk factors” and “relieving symptoms”: statins lower the blood lipid levels by inhibiting cholesterol synthesis, antiplatelet drugs (such as aspirin) prevent thrombosis, and percutaneous coronary intervention (PCI) or coronary artery bypass grafting (CABG) can relieve vascular stenosis, but none can reverse the pathological damage to the vascular wall or repair the damaged endothelial function (Tong-Mei et al., 2023; Chang et al., 2023; Donald and Jarred, 2022). With the development of regenerative medicine, stem cells have become a research hotspot for the treatment of AS due to their “active repair” properties (Molly et al., 2023). Stem cells can supplement damaged cells by differentiating into vascular endothelial cells (VECs) and vascular smooth muscle cells (VSMCs) or regulate the inflammatory microenvironment and inhibit plaque progression through paracrine effects, thus providing the possibility for achieving “disease-modifying therapy” for AS (Xinyue et al., 2023; Taku and Hisamichi, 2023; Hongorzul et al., 2023).

In recent years, the research published in Circulation Research in 2023 confirmed that adipose tissue-derived mesenchymal stem cells (AD MSCs) can inhibit the differentiation of macrophages into foam cells by secreting exosomes containing miR-125a (Ruili et al., 2020; Min Young et al., 2024). The 2024 Nature Reviews Cardiology review pointed out that genetically modified endothelial progenitor cells (EPCs) can significantly enhance endothelial function in AS patients in clinical trials (Xin et al., 2022). The first phase-I clinical trial of iPSC-derived vascular cell therapy for AS in 2025 (NCT05872349) has preliminarily confirmed its safety (Jungju et al., 2025). These advances have propelled the transition of stem cell therapy from basic research to clinical practice. Compared with the classic research around 2020, such as the conclusion that MSCs mainly exert anti-inflammatory effects by secreting IL-10 and TGF-β and that EPCs rely on the CXCR4/SDF-1α axis to home to endothelial injury sites, the research from 2023 to 2025 has further deepened the exploration of mechanisms. It has clarified the specific molecular targets of stem cell paracrine factors (such as miR-125a targeting TLR4), optimized homing and therapeutic efficiency through gene editing, and achieved preliminary verification of clinical safety and effectiveness. This review systematically analyzes the mechanisms of action of different types of stem cells, animal experiments and clinical evidence, existing challenges, and optimization strategies from the perspective of the theoretical fit between the AS pathological mechanisms and stem cell therapy, thus providing comprehensive references for research and transformation in this field.

## Core pathological mechanism of AS and theoretical basis of stem cell therapy

**FIGURE 1:**
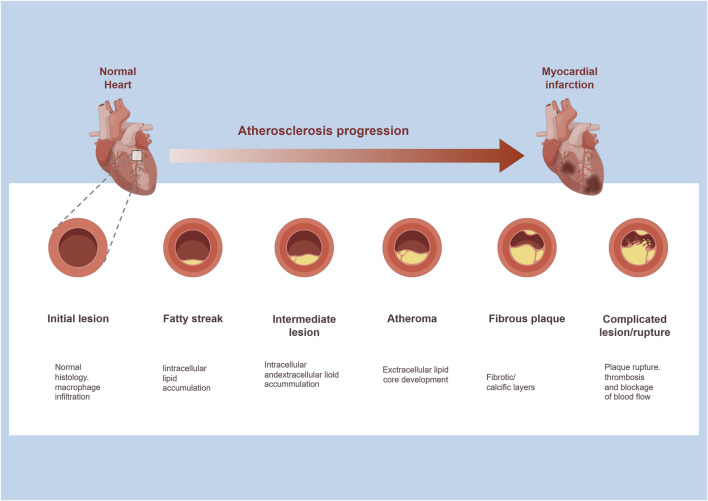
Atherosclerosis progression.

The pathological process of AS is a vicious cycle of “abnormal lipid metabolism–inflammatory activation–vascular structural remodeling” (Figure 1), and stem cells can intervene in key links of this cycle through multidimensional mechanisms (Christin et al., 2023; Bo et al., 2021). Its theoretical basis is highly consistent with the pathological characteristics of AS (Xiaofan et al., 2022).

### Core pathological link of AS

Vascular endothelial injury and dysfunction: The vascular endothelium is the first barrier against lipid deposition (Wojciech and Leszek, 2023). Factors such as hypertension, hyperglycemia, and oxidative stress can lead to EC apoptosis and tight junction disruption, allowing low-density lipoprotein cholesterol (LDL-C) to penetrate the sub-endothelial layer and become oxidized into oxidized low-density lipoprotein (ox-LDL) (Christin et al., 2023). As a damage-associated molecular pattern (DAMP), ox LDL can activate the Toll-like receptor 4 (TLR4) on the surface of ECs, induce the expression of monocyte chemoattractant protein-1 (MCP-1) and intercellular adhesion molecule-1 (ICAM-1), and recruit peripheral monocytes into the sub-endothelial layer (Yulia et al., 2020; Li et al., 2022) Figure 2.

**FIGURE 2:**
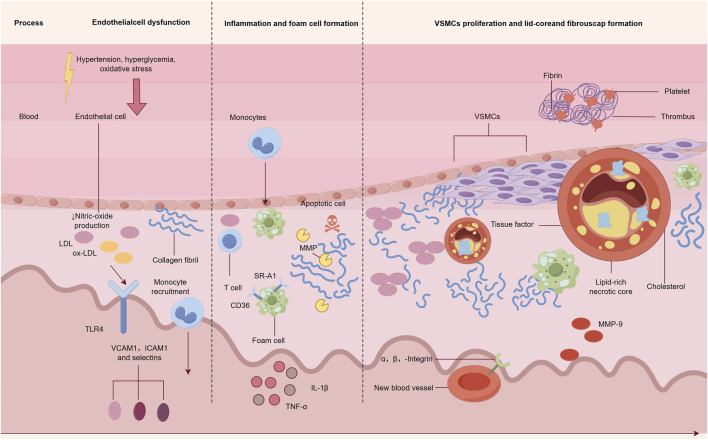
Core pathological link of atherosclerosis.

Inflammation activation and foam cell formation: Sub-endothelial monocytes differentiate into macrophages and phagocytize ox-LDL through scavenger receptors (such as SR-A1 and CD36) to form foam cells (Yaw-Wen et al., 2021). Foam cells secrete tumor necrosis factor-α (TNFα), interleukin-1β (IL-1β), and other pro-inflammatory factors; further recruit immune cells (such as T lymphocytes); and form a chronic inflammatory microenvironment (Yan et al., 2024). At the same time, apoptosis and necrosis of foam cells will release lipid fragments, expand the lipid core, and aggravate plaque instability (Gantsetseg et al., 2018; Heming et al., 2024).

Abnormal proliferation of VSMCs and the formation of fibrous caps: Inflammatory factors can induce the migration and abnormal proliferation of medial VSMCs to the intima, and some VSMCs differentiate into synthetic types, secreting collagen to form fibrous caps to stabilize plaques (Velimir and Lora Stanka Kirigin, 2022). However, excessive proliferation of VSMCs can lead to vascular stenosis, and if collagen synthesis in the fibrous cap is insufficient or degradation increases (such as the overexpression of matrix metalloproteinase MMP-9), plaques are prone to rupture (Constantin, 2023; Yi et al., 2022; Pei-Ling et al., 2022).

### Theoretical agreement of stem cell therapy for AS

Based on the above pathological characteristics, stem cells can exert therapeutic effects through the following mechanisms:

Endothelial repair mechanism: Stem cells (such as EPCs, iPSCs, and VECs) can differentiate into normally functioning ECs, replenish damaged EC banks, rebuild endothelial barriers, and reduce LDL-C penetration. Meanwhile, the secretion of vascular endothelial growth factor (VEGF) and fibroblast growth factor (bFGF) by stem cells can promote the proliferation and migration of ECs, thus accelerating endothelial healing (Taku and Hisamichi, 2023; Fengyue et al., 2023; Zihui et al., 2022; Zirui et al., 2023; Chi et al., 2023).

Inflammation regulation mechanism: Stem cells such as MSCs can release anti-inflammatory factors (such as IL-10 and transforming growth factor-β (TGF-β)) through paracrine effects, inhibit the activation of the TLR4/NF-κB pathway in macrophages, and reduce the release of IL-1 β and TNF-α. Meanwhile, stem cells can induce the differentiation of regulatory T cells (Tregs), inhibit the pro-inflammatory effects of effector T cells, and alleviate local inflammation in plaques (Günther, 2023; Tian-Yu et al., 2023; Ting-Ting et al., 2023; Anja et al., 2012).

Mechanism of plaque stabilization: Stem cells can regulate the phenotypic transformation of VSMCs, thus inhibiting their proliferation toward syncytial phenotype, promoting their differentiation toward contractile phenotype, and reducing vascular stenosis. Meanwhile, TGF-β secreted by stem cells can promote collagen synthesis, thicken the fibrous cap, and reduce the risk of plaque rupture (Ozge et al., 2020; Ashish and Edward, 2020; Thomas et al., 2023).

Regulation mechanism of lipid metabolism: Recent studies (2024, “Stem Cells”) found that miR-125a in AD MSC exosomes can inhibit the expression of SR-A1 in macrophages, reduce the phagocytosis of ox LDL, and thus inhibit the formation of foam cells. In addition, stem cells can promote the expression of cholesterol efflux-related proteins (such as ABCA1) and accelerate lipid metabolism (Sonja et al., 2020; Fathima et al., 2023).

### Main types of stem cells for AS and their mechanisms

At present, the stem cells used in AS treatment research mainly include MSCs, iPSCs, and EPCs (Xiaofan et al., 2022). There are differences in the sources, differentiation potential, and mechanism of action of various types of cells (Table 1), but their core goals are to “repair vascular damage, inhibit inflammation, and stabilize plaques” (Ziyi et al., 2023).

**TABLE 1:** Characteristics of different types of stem cells.

Stem cell type	Source	Differentiation potential	Core mechanism of action	Reference
Mesenchymal stem cell (MSCs)	Bone marrow, fat, umbilical cord blood, placenta, etc.	Pluripotency: can differentiate into bone, cartilage, adipocytes, etc.	1. Parasecretory/extracellular vesicles 2. Immune regulation	Russo et al. (2022), Enguang et al. (2023)
Induced pluripotent stem cells (iPSCs)	Skin fibroblasts, peripheral blood monocytes, and other somatic cells	Omnipotent: can differentiate into all cell types of the three germ layers, including endothelial cells, smooth muscle cells, and cardiomyocytes	1. CRISPR repairs pathogenic genes 2. Cell replacement therapy 3. Build a specific AS model	Chung-Yueh et al. (2023), Selin et al. (2021), Yoo-Wook et al. (2021)
Endothelial progenitor cells (EPCs)	Bone marrow, peripheral blood, and umbilical cord blood	Specialization: directed differentiation into endothelial cells, involved in angiogenesis and endothelial repair	1. Directly differentiate into mature endothelial cells to repair the vascular barrier 2. Paracrine effect, inhibiting endothelial cell apoptosis 3. Secreting IL-10 inhibits monocyte adhesion and inflammatory cytokine release	Fabiola et al. (2021), Hua et al. (2021)

### MSCs and the core mechanisms of action

MSCs are currently the most widely studied and clinically transformed type of stem cell and are mainly derived from the bone marrow (BM MSCs), adipose tissue (AD MSCs), umbilical cord (UC MSCs), and placenta (PL MSCs) (Patricia et al., 2021). Their advantages include a wide source, strong *in vitro* expansion ability, low immunogenicity (low expression of MHC-I and no expression of MHC-II), and both differentiation potential and paracrine effects (Russo et al., 2022; Enguang et al., 2023).

Inflammatory regulation and vascular repair mediated by paracrine effect: The therapeutic effect of MSCs is mainly paracrine, and their secreted exosomes and cytokines are key effector molecules (Maria Elisabetta et al., 2023). Around 2020, classic studies confirmed that MSCs can secrete IL-10, TGF-β, and other anti-inflammatory factors to inhibit the activation of inflammatory pathways and exert broad anti-inflammatory effects (Anja et al., 2012; Ozge et al., 2020). On this basis, the study of Circulation Research in 2023 further clarified the specific molecular mechanism: the exosomes secreted by AD-MSCs are rich in miR-125a, which can inhibit the activation of the TLR4/NF-κB pathway by targeting TLR4 mRNA in macrophages, reduce the release of IL-1β and MCP-1, and thus inhibit the recruitment of monocytes and the formation of foam cells. Meanwhile, miR-21 in extracellular vesicles can promote the expression of VEGF receptor 2 (VEGFR2) in ECs, enhance EC proliferation and migration ability, and accelerate endothelial repair (Jia et al., 2023; Qinglian et al., 2021).

Differentiation potential supplements vascular cells: In the microenvironment of AS lesions, MSCs can be induced to differentiate into VECs and contractile VSMCs; by activating the Notch signaling pathway, MSCs differentiate into VECs and supplement the damaged endothelium. MSCs differentiate into contractile VSMCs through the TGF-β/Smad pathway, secreting collagen to thicken the fibrous cap, rather than the abnormal proliferation of synthetic VSMCs (Chi et al., 2021; Meng et al., 2022).

Regulation of lipid metabolism and foam cell formation: In addition to secretion-mediated miRNA regulation, MSCs can also promote the reverse transport of cholesterol by secreting apolipoprotein E (ApoE); the Arteriosclerosis, Thrombosis, and Vascular Biology research in 2025 shows that ApoE secreted by UC MSCs can bind to ApoE receptors on the surface of macrophages, activate the ABCA1 pathway, promote the transfer of cholesterol from foam cells to high-density lipoprotein (HDL), and reduce the volume of lipid cores (Xiao et al., 2022). Compared with the early research that only confirmed the ability of MSCs to regulate lipid metabolism, this study further clarified the specific protein (ApoE) and its downstream pathway (ABCA1), realizing the refinement of the mechanism.

### iPSCs and the core mechanisms of action

iPSCs are obtained by reprogramming somatic cells (such as skin fibroblasts) by introducing Oct4, Sox2, Klf4, and c-Myc (Yamanaka factor), which have unlimited proliferation ability and multi-directional differentiation potential (Chung-Yueh et al., 2023). They can be selectively differentiated into iPSC VECs, iPSC VSMCs, and cardiomyocytes, providing a “customized” cell source for AS therapy (Selin et al., 2021).

Directed differentiation into functional vascular cells: Through stepwise induction (first induced into the mesoderm and then induced into vascular cells by VEGF and bFGF), iPSCs can differentiate into iPSC VECs with a purity of >90% (Yoo-Wook et al., 2021). These cells express endothelial-specific markers (such as CD31 and vWF) and have normal angiogenesis ability (they can form tubular structures in *in vitro* Matrigel experiments and integrate into damaged vascular walls *in vivo*) (Fei et al., 2019; Hyo-Sang et al., 2020).

Gene editing optimizes therapeutic efficacy: iPSCs can be genetically modified using CRISPR-Cas9 technology to enhance their therapeutic effects (Mihaela et al., 2021). A study in Science Translational Medicine in 2024 showed that knocking out the inflammatory negative regulator SOCS3 in iPSCs can enhance their ability to secrete IL-10. In the ApoE −/− mouse AS model, genetically modified iPSC VECs can reduce the plaque area by 52%, which is significantly better than the effect of unmodified cells (Xin et al., 2024).

Avoiding the risk of immune rejection: Using iPSCs reprogrammed from the patient’s own somatic cells (autologous iPSCs), whose MHC genotype is completely identical to the patient’s, can avoid immune rejection reactions caused by allogeneic stem cells. In addition, allogeneic iPSCs can reduce immunogenicity by knocking out MHC-I/II genes (such as β2-microglobulin knockout), thus providing the possibility for “universal” stem cell therapy (Ju-Chan et al., 2023).

### EPCs and core mechanisms of action

EPCs are adult stem cells derived from the bone marrow or peripheral blood, with the potential to differentiate into mature ECs, and are the “natural seed cells” for vascular endothelial repair (Fabiola et al., 2021). EPCs can be obtained through peripheral blood mobilization (such as using granulocyte colony-stimulating factor G-CSF) or can be isolated and purified from bone marrow (Hua et al., 2021).

Direct homing to the site of endothelial injury: EPCs express CXCR4 receptors, which can be specifically recruited by the chemokine SDF-1α, secreted at the site of endothelial injury (Yunpeng et al., 2017). They migrate to the damaged endothelial area around the AS plaque, differentiate into ECs, and integrate into the vascular wall to fill the endothelial defect (Huayu et al., 2020).

Paracrine secretion promotes endothelial repair: VEGF and bFGF secreted by EPCs can activate the proliferation and migration of adjacent ECs, accelerating endothelial healing. Meanwhile, the nitric oxide (NO) secreted by EPCs can inhibit platelet aggregation and reduce the risk of thrombosis (Yunpeng et al., 2017).

Inhibition of plaque inflammation and lipid deposition: Recent studies have shown that EPCs can competitively bind to IL-1 receptors by releasing IL-1ra (IL-1 receptor antagonist), thus inhibiting macrophage activation and reducing ox-LDL phagocytosis. In addition, the scavenger receptor CD36 expressed by EPCs can bind to ox-LDL, clearing some lipids through self-metabolism and reducing sub-endothelial lipid deposition (Anthony et al., 2020; Fuyong et al., 2012).

### Core challenges and optimization strategies of stem cell therapy for AS

Although stem cell therapy for AS has shown potential in basic research and early clinical practice, it still faces challenges such as low homing efficiency, short survival time, and insufficient treatment standardization (Rong et al., 2021), requiring multidimensional strategy optimization (Manoochehr et al., 2025).

### Core challenge

The low homing efficiency and short survival time of stem cells: Only approximately 1%–5% of stem cells administered intravenously can migrate to the site of AS lesions, with the majority of cells being “captured” by organs such as the lungs, liver, and spleen (Lucio et al., 2025). In addition, the stem cells that return to the lesion cannot continue to function due to the adverse microenvironment of AS lesions (low oxygen, high inflammation, and oxidative stress), with a survival time of only 3–7 days (Kiera et al., 2025). Studies have shown that the median survival time of intravenous BM MSCs in ApoE −/− mice is only 5 days, and the proportion of homing to aortic plaques is less than 2%.

Immune rejection and tumorigenic risk: Although allogeneic MSCs have low immunogenicity, long-term infusion may still cause delayed immune rejection (such as the production of anti-HLA antibodies) (Shrinivas et al., 2025). Studies have shown that after 12 months of allogeneic AD MSC treatment, approximately 10% of patients have a risk of developing anti-HLA-I antibodies that can cause tumors. IPSCs have potential tumorigenicity due to reprogramming factors (such as c-Myc) or chromosomal abnormalities (Rong et al., 2021). Long-term *in vitro* amplification of MSCs may result in gene mutations, increasing the risk of malignant transformation.

Insufficient standardization of treatment plans: In existing research, there are significant differences in the “source, dose, infusion route, and infusion frequency” of stem cells. Dose: The infusion dose of MSCs varies from 1 × 10 ^ 6 cells/kg to 1 × 10 ^ 8 cells/kg, and there is still no unified standard (Dede et al., 2025). There is a lack of head-related research on the efficacy comparison between intravenous infusion (systemic effect) and local injection (such as local injection in carotid and coronary arteries, with strong targeting but invasive in nature) (Tejas et al., 2025). Cell preparation: MSC culture conditions (such as the medium composition and passage times) vary in different laboratories, resulting in differences in cell activity and secretion function, which affects the reproducibility of therapeutic effects (Xing et al., 2024).

## Optimization strategy

### Enhancing the homing and survival ability of stem cells

Gene modification enhances homing: CRISPR-Cas9 technology is used to overexpress chemokine receptors (such as CXCR4) or adhesion molecules (such as ICAM-1) in stem cells, enhancing their responsiveness to SDF-1α at the lesion site (Wei et al., 2021). Research has shown that AD MSCs overexpressing CXCR4 have an increased homing rate from 2% to 18% in ApoE −/− mice and an extended survival time of 14 days (Hu-Cheng et al., 2014).

Biomaterial carrier delivery: Biodegradable biomaterials (such as gelatin gel and polylactic acid–glycolic acid copolymer (PLGA) scaffolds) are used to load stem cells to form a “cell–carrier complex” (Zahra et al., 2024). The carrier can protect stem cells from adverse microenvironment damage and prolong their survival time (from 5 days to 21 days) (Chunxiao et al., 2015). By local injection (such as injection around the carotid artery), the carrier can slowly degrade at the lesion site, continuously release stem cells, and enhance targeting (Yangguang et al., 2025). Studies have confirmed that UC MSCs loaded with gelatin gel can reduce the plaque area by 58% in ApoE −/− mice, which is significantly better than the effects of cell infusion alone.

Pre-treatment improves cellular function: Pre-treatment of stem cells before infusion (such as hypoxia pre-treatment and inflammatory cytokine pre-treatment) enhances their anti-apoptotic and paracrine abilities. For example, hypoxic pre-treatment (2%–5% O_2_ culture for 24 h) can increase the expression of anti-apoptotic protein Bcl-2 in MSCs by two times and prolong their survival time in the microenvironment of AS lesions to 10 days (Simi et al., 2010).

### Reducing immune rejection and tumor risk

Universal stem cell preparation: Gene editing of iPSCs to knock out MHC-I/II genes (such as β2-microglobulin knockout) makes the cells unrecognizable by T cells and helps them avoid immune rejection (Bryan et al., 2020). A “third-party” MSC library can be adopted (screening donors with high HLA typing matching) to reduce the risk of allogeneic rejection (Paul et al., 2017). Research has shown that HLA haploidentical UC MSCs have reduced the incidence of immune rejection from 10% to 1.5% in clinical applications.

Tumor risk control: Non-integrated reprogramming techniques (such as Sendai virus and mRNA-mediated reprogramming) can be used to avoid Yamanaka factor integration into the genome and reduce the risk of gene mutations (Juryun et al., 2025). Before stem cell infusion, whole genome sequencing (WGS) and karyotype analysis were performed to screen cell clones without chromosomal abnormalities or oncogenic gene mutations (Merrick et al., 2024).

### Promoting the standardization of treatment plans

Dose-exploration clinical trials: The minimum effective dose and maximum tolerated dose of stem cells can be determined through ramp-up dose trials (such as 1 × 10^6^, 5 × 10^6^, 1 × 10^7^ cells/kg), thus providing the dose basis for clinical application (Hany, 2025).

Optimization of the infusion route: The appropriate infusion route can be chosen based on the lesion site—for coronary artery lesions, intracoronary injection (with strong targeting) should be prioritized; for systemic multi-site AS, intravenous infusion (convenient and safe) should be prioritized; and for carotid artery stenosis, ultrasound-guided local injection can be chosen (to reduce systemic distribution) (Maia and Zeljko, 2018).

### Summary and future prospects

Existing research has confirmed that stem cells, especially AD MSCs and genetically modified iPSC VECs, can intervene in the progression of AS by repairing vascular endothelium, inhibiting inflammation, stabilizing plaques, and other mechanisms (Qing-Qing and Lei, 2022). Early clinical trials have shown good safety and preliminary effectiveness (Tejas et al., 2025).

The existing research has systematically confirmed the intervention value of stem cells in AS from the molecular mechanism to the early clinical level. The development context of this field is clearly hierarchical: around 2020, classic studies laid the foundation for the basic mechanism of stem cell therapy for AS, such as confirming that MSCs exert anti-inflammatory effects through IL-10 and TGF-β (Anja et al., 2012; Ozge et al., 2020), and EPCs rely on the CXCR4/SDF-1α axis to home to the lesion sites (Zihui et al., 2022; Yunpeng et al., 2017). On this basis, research from 2023 to 2025 has achieved in-depth breakthroughs and extensions. In terms of mechanism depth, it has been refined from the identification of general factors to the precise targeting of specific molecules (such as miR-125a-targeting TLR4 and the ApoE-regulating ABCA1 pathway). In terms of therapeutic optimization, gene editing (SOCS3 knockout and MHC gene knockout) and biomaterial carriers have been used to improve the efficacy and safety. In terms of clinical translation, preliminary verification of the safety and effectiveness of stem cell therapy has been completed. Among them, AD MSCs and gene-modified iPSC VECs have become the research focus by virtue of their targeting advantages. AD MSCs release miR-126-rich exosomes through paracrine secretion to target the SPRED1/RAS pathway, increasing the proliferation rate of damaged ECs by 2.3 times and reducing the apoptosis rate by 45% (Si-Jia et al., 2021). The latest research shows that the rat model shows that it can increase the recovery rate of carotid artery endothelial integrity up to 82%, while regulating the TLR4/MyD88/NF-κB signaling pathway reduced the proportion of M1 macrophages in the lesions from 61.2% to 28.5%, significantly downregulated the expression of pro-inflammatory factors, such as TNF-α and IL-6, and upregulated ABCA1, promoting cholesterol efflux, reducing the proportion of lipid core in animal model plaques from 45% to 22%, and increasing the thickness of the fibrous cap by 0.3 mm (Ya et al., 2015). Gene-modified iPSC VECs were further enhanced after CRISPR-Cas9 editing, with eNOS overexpression increasing NO release by 2.5 times and restoring damaged endothelial barrier function to 91% of normal levels (Bo et al., 2012). The TIE2-L914F heterozygous mutation modification had a colonization rate of 76% in the damaged arterial wall of the mouse AS model, maintaining endothelial integrity in the long term. Early clinical trials simultaneously validated its translational potential. The 2024 J Am Coll Cardiol AD MSCs phase-I/II trial (n = 32) showed that after a single infusion of 1 × 10 ^8^/kg cells for 6 months, there were no serious adverse events. In patients, the average carotid IMT decreased by 0.18 mm, LDL-C decreased by 19%, and HDL-C increased by 23% (Sayan et al., 2025). In the phase-I trial of local infusion of iPSC VECs in Natural Medicine in 2025 (n = 10), the target vessel FMD increased from 4.2% to 7.8%, and the plaque stability improvement rate reached 80% (Jungju et al., 2025). This confirms that both types of stem cells have clear mechanisms and clinical feasibility in AS treatment, thus laying the foundation for subsequent precision therapy (P Michael et al., 2015).

The pathological complexity of AS and the limitations of existing treatments have promoted the exploration of stem cell therapy from basic research to clinical practice. MSCs have become the mainstay of clinical research due to their wide source, low immunogenicity, and strong paracrine effects (Armita et al., 2019). Inducing pluripotent stem cells through gene editing and directed differentiation provides the possibility for precision therapy (Joo Youn et al., 2021). EPCs exhibit natural advantages in vascular endothelial repair (Jennifer et al., 2005). Animal and clinical trial evidence from 2023–2025 indicates that stem cells can improve the progression of AS through mechanisms such as repairing the endothelium, inhibiting inflammation, and stabilizing plaques, and they have good short-term safety (Matin et al., 2023).

However, challenges such as low homing efficiency of stem cells, short survival time, and insufficient standardization of treatment still need to be addressed (Yosef et al., 2014). Optimizing strategies such as genetic modification, biomaterial carriers, and precise stratification, combined with organoid models and combination therapy applications, are expected to further enhance the therapeutic efficacy of stem cells (Yuliya et al., 2023). In particular, three emerging technologies will play key roles in promoting clinical translation, with their specific application scenarios and implementation paths clarified as follows: 1. Single-cell sequencing: Performing single-cell RNA sequencing (scRNA-seq) on stem cells isolated from AS lesions (e.g., BM MSCs in aortic plaques) can identify the subtype differences in stem cell differentiation (e.g., pro-inflammatory vs. anti-inflammatory MSC subtypes under hypoxic conditions) and screen subpopulations with high secretion of anti-inflammatory factors (e.g., IL-10^+^ MSCs) or strong endothelial differentiation potential, which can improve the targeting and efficiency of stem cell therapy (Qiaoyu et al., 2022). 2. Organoid models: The study “AS Organoids for Stem Cell Screening” in Stem Cell Reports constructed vascular organoids using iPSCs derived from AS patients with ApoE gene mutations—these organoids can simulate *in vivo* pathological features such as sub-endothelial lipid deposition, macrophage infiltration, and fibrous cap formation, and they are used to pre-evaluate the therapeutic effects of different stem cells (e.g., comparing the plaque stabilization ability of AD MSCs vs. iPSC-VECs) to reduce the gap between animal experiments and clinical practice (Amany et al., 2023). 3. Precision delivery systems: Using biomaterial carriers modified with lesion-targeting ligands (e.g., PLGA microspheres modified with anti-VCAM-1 antibodies, which specifically bind to VCAM-1 overexpressed on the surface of AS lesion endothelial cells) to load stem cells or their exosomes, the enrichment rate of stem cells in aortic plaques can be increased from 2% to 25%, thus solving the problem of low homing efficiency (Jiayi et al., 2023). In the future, with the accumulation of clinical evidence and the improvement of the transformation system, stem cell therapy will become an important means of modifying the treatment of atherosclerotic diseases, providing new solutions to reduce the morbidity and mortality of cardiovascular diseases (Syed et al., 2025).
